# Shared Neural Substrates Underlying Reading and Visual Matching: A Longitudinal Investigation

**DOI:** 10.3389/fnhum.2020.567541

**Published:** 2020-10-22

**Authors:** Xin Cui, Zhichao Xia, Catherine McBride, Ping Li, Jinger Pan, Hua Shu

**Affiliations:** ^1^State Key Laboratory of Cognitive Neuroscience and Learning and IDG/McGovern Institute for Brain Research, Beijing Normal University, Beijing, China; ^2^School of Systems Science, Beijing Normal University, Beijing, China; ^3^Department of Psychology, Brain Mind Institute, The Chinese University of Hong Kong, Hong Kong, China; ^4^Department of Chinese and Bilingual Studies, Faculty of Humanities, The Hong Kong Polytechnic University, Hong Kong, China; ^5^Department of Psychology, The Education University of Hong Kong, Hong Kong, China

**Keywords:** left fusiform gyrus, longitudinal, Chinese, print-to-sound mapping, Visual Matching, reading

## Abstract

The role of visual skills in reading acquisition has long been debated and whether there is shared neurobiological basis between visual skills and reading is not clear. This study investigated the relationship between Visual Matching and reading and their shared neuroanatomical basis. Two hundred and ninety-three typically developing Mandarin-speaking children were followed in a longitudinal study from ages 4 to 11 years old. A subsample of 79 children was further followed up at 14 years old when the MRI data were collected. Results showed that the development of Visual Matching from ages 6 to 8 predicted reading accuracy at age 11. In addition, both the development of Visual Matching and reading accuracy were associated with cortical surface area of a cluster located in fusiform gyrus. These findings suggested that the mapping from visual codes to phonological codes is important in learning to read and that left fusiform gyrus provided neural basis for such mapping. Implications of these findings in light of a new approach toward the neurocognitive mechanisms underlying reading development are discussed.

## Introduction

Reading is a complex skill that involves processing written words and the conversion from visual forms to speech. Previous studies have shown that dyslexic readers have more problems with processing alphanumeric stimuli (e.g., letters, digits), than non-alphanumeric stimuli (e.g., symbols, colors, objects, dice surfaces); the former stimuli are verbally codable whereas the latter do not (directly) map onto phonological codes (Ziegler et al., 2010; Pan et al., 2013). However, it is so far unclear with regard to the role of such mapping between visual forms and phonological codes of alphanumeric stimuli in reading among typically developing children. Furthermore, it is unclear whether this mapping also plays an important role in non-alphabetic scripts such as Chinese, and what the neurobiological basis is. The present study sets out to investigate these issues by comparing the contributions of two visual tasks that differ in terms of phonological activation of the stimuli, i.e., Cross Out (Woodcock and Johnson, 1989), a task that involves no phonological activation for which participants have to visually process non-alphanumeric stimuli and no phonological coding is involved, and Visual Matching (Woodcock and Johnson, 1989), a task that involves visual processing of alphanumeric stimuli that can be mapped to sound. A systematic comparison of these tasks measured annually from ages 6 to 8 and their underlying neurocognitive mechanisms measured at age 14 enables us to address critical issues of reading development in Chinese-speaking children.

During reading, readers have to visually process words and map them to their phonological representations. In the early stage of reading acquisition, children’s brains show some tendency to process words as if they are pictures (the “logographic stage”; Ehri and McCormick, 1998). Visual skills as early as in kindergarten 1 (K1-ages 3 to 4 in some places in Asia) are able to predict word reading in K2 (roughly ages 4 to 5) (Lin et al., 2016). However, as children grow older and rely more on decoding in reading, visual skills do not seem to be as important as in the early stage (Yang et al., 2013; Pan et al., 2020).

The importance of visual skills in reading may be related to orthography-phonology correspondence as activation of orthographic representations of the words apparently requires some visual processing. Building print-to-sound associations is one of the most important prerequisites in reading acquisition (Rosenthal and Ehri, 2008). In a variety of reading-related tasks, processing of graphemic stimuli are most closely associated with literacy skills. For example, among typically developing children, rapid naming tasks using letters and digits as stimuli predict reading ability better than rapid naming tasks using colors and objects (e.g., McBride-Chang, 1996; Schatschneider et al., 2004; Bowey et al., 2005). This is also supported by evidence from studies on dyslexic readers that they differ more from typically developing children on tasks involving alphanumeric stimuli (e.g., letters and digits) than non-alphanumeric stimuli (e.g., colors, objects, and symbols), irrespective of whether the tasks require explicitly phonological activation of the visual stimuli (Ziegler et al., 2010; Pan et al., 2013; Bakos et al., 2017). Moreover, children with better integration of print-to-sound mapping have a better reading performance than their peers (McBride-Chang, 1996; Cardoso-Martins and Pennington, 2004; Schatschneider et al., 2004; Pan et al., 2013). Together, these findings suggest that visual processing itself does not predict reading independently.

To better understand reading development, researchers have been investigating the neurobiological basis of the print-to-sound mapping process. The left fusiform gyrus, including the visual word form area (VWFA) and surrounding cortex, has long been implicated in the processing of visual words (Glezer et al., 2009; Dehaene and Cohen, 2011; Hirshorn et al., 2016). This so-called “visual word form system” (VWFS) involves VWFA and is inherently coupled with the higher-order language system at both functional and structural levels (Stevens et al., 2017; Chen et al., 2019). Meanwhile, previous studies have shown that activation in this area is correlated with phonological awareness, suggesting that when processing print, this area is automatically involved – even if no explicit phonological processing is required – in the phonological coding system (Frost et al., 2009; Price and Devlin, 2011; Pugh et al., 2013). Individuals with impaired print-to-sound mapping ability (e.g., developmental dyslexia) often show reduced functional connectivity between the VWFA and other language related regions in the left frontal and parietal lobes (van der Mark et al., 2011). Moreover, the emergence of VWFS relies on its connectivity to the language system, which is formed before reading instruction (Saygin et al., 2016) and it develops rapidly through intensive learning of print and corresponding sounds (Brem et al., 2010; Dehaene-Lambertz et al., 2018). In sum, evidence from functional neuroimaging studies suggests that the VWFS serves as an interface between the visual and phonological systems, which underpins the conversion of print to correspondent sounds.

However, compared to the large number of functional neuroimaging studies on the VWFS (e.g., Brem et al., 2010; Bach et al., 2013; Dehaene-Lambertz et al., 2018), fewer studies have examined its structure. Furthermore, most previous studies have relied on a comparison between children who were either dyslexic or who had a family risk of dyslexia as compared to typically developing children (Raschle et al., 2011; Skeide et al., 2016; Beelen et al., 2019; Kuhl et al., 2020). While these group comparisons provide evidence for the neural basis underlying reading difficulties, little is known about whether the properties of neuroanatomy are also associated with individual differences in reading and print-to-sound mapping abilities, especially in the very early stages when children are just starting to learn printed words systematically (e.g., early primary school) and in a non-alphabetic script.

Additionally, previous research investigating the role of the left fusiform gyrus in reading development focused mainly on gray matter volume which is the product of cortical thickness and surface area. However, cortical thickness and surface area are different measures of brain morphometry and offer separate indices for neurodevelopment and diseases (Panizzon et al., 2009; Winkler et al., 2018), and correlate with different cognitive abilities (Noble et al., 2015; Vuoksimaa et al., 2015). On the one hand, the surface area is thought to be more related to prenatal factors and to have a cascading effect on cognitive abilities (Raznahan et al., 2012; Walhovd et al., 2012). Cortical thickness, on the other hand, is probably more sensitive to the environment and to the developmental changes of cognitive ability over time (Sowell et al., 2004; Jha et al., 2019). Evidence for a significant association between reading ability and surface-based measures of the fusiform gyrus mainly comes from dyslexia research (Frye et al., 2010; Altarelli et al., 2013; Hosseini et al., 2013; Qi et al., 2016; Williams et al., 2018). Studies have also shown that surface area may be relatively steady after birth and show long-lasting impact on reading and/or reading-related cognitive abilities (Raznahan et al., 2011; Striedter et al., 2015; Williams et al., 2018). Nevertheless, whether there is such an enduring relationship between brain morphometry and individual differences in reading-related abilities among typically developing children remains unclear.

A number of tasks have been used by researchers to examine children’s visual abilities. Visual Matching and Cross Out are two tasks that involve speeded visual processing. While Cross Out requires children to process non-codable symbols, Arabic digits have been used as stimuli in Visual Matching. Although Visual Matching does not explicitly require children to activate phonological representations of the digits, they might nevertheless be activated automatically, thus may reflect the process of visual-verbal conversion in reading. Recently, Pan et al. (2020) tested the relation between word reading and Cross Out/Visual Matching among Chinese children from 6 to 8 years old. They showed that while Visual Matching at age 6 was able to predict word reading at age 7, Cross Out did not contribute to word reading. Such findings support the importance of print-to-sound mapping in the course of reading development. However, that study tested the cross-lag relationships of reading with the Cross Out and Visual Matching tasks. In the present study, we sought to determine whether Visual Matching would also emerge as important for reading acquisition beyond the initial stages of reading. After all, Chinese is an opaque orthography, meaning that the print-to-sound mappings are largely arbitrary. As children enter primary school, the acquisition of print-to-sound mappings grows rapidly as more characters are learned. If this Visual Matching ability is useful for reading in Chinese over time, this may suggest a unique approach to identifying and remediating difficulties in reading.

In the present longitudinal study, using the Cross Out task as a comparison to Visual Matching, we tested the role of print-to-sound mapping as reflected in Visual Matching (but not Cross Out) in reading development and its shared neurobiological basis with reading. Visual Matching and Cross Out were tested annually when children were 6 to 8 years old. As for the dependent variable, we focused on word reading ability which served as a basis for the neurobiological model of reading and has been widely used in neuroimaging studies to explore the development and impairments of neural circuitry of reading (Pugh et al., 2001; Taylor et al., 2013; Dehaene-Lambertz et al., 2018). The study reported here was part of a large-scale longitudinal study initiated some 20 years ago. At that time research-dedicated MRI scanners were rare in China for collecting neuroimaging data. The current study was only able to collect brain morphometry measures of the children at a later time and we therefore tested the association of early predictors of reading with the later acquired brain data (when the children became adolescents). This is a rather different approach from previous studies that have used concurrent or earlier brain measures to predict reading performance (e.g., Hoeft et al., 2011; Bach et al., 2013; Kuhl et al., 2020).

Specifically, based on the extant literature, we asked in the present study (a) whether the initial ability to convert visual forms to phonological codes reflected by Visual Matching or its growth rate would emerge important for reading performance in a later stage; and (b) whether the visual-verbal conversion and reading share neurobiological basis, especially in the left fusiform gyrus, as it has been shown to be related to higher-order linguistic processing. Given that the rapid development of the VWFS only emerges after formal instruction in reading (Brem et al., 2010; Dehaene-Lambertz et al., 2018), we predicted that the growth rate of Visual Matching would play a more important role in reading development than its initial status as participants’ Visual Matching was first measured at age 6 before they received formal education. In addition, as print-speech mapping becomes extremely important after formal reading instruction, we predicted that the cortical morphometry of the left fusiform would be associated with individual differences in growth rate of Visual Matching.

## Materials and Methods

### Participants

Two hundred ninety-three children were selected from a longitudinal cohort that had initially participated in the study of the development of Chinese Communicative Development Inventory (Tardif et al., 2008). These children were originally recruited from maternal-child health care service centers in Beijing. All participating children had a normal IQ. According to their health care records, they had no diagnosed difficulties in any aspects including mental, sensory, and physical domains. They were followed and tested roughly every 12 months until age 11. Children started primary school after the assessment at age 6 (*M* = 6.42 years old, *SD* = 0.30). A subsample of 79 children participated in the assessment consisting of behavioral and magnetic resonance imaging (MRI) sessions at age 14. The current study focused on the Cross Out and Visual Matching tasks at ages 6, 7, and 8, and character recognition task at age 11. Non-verbal IQ, vocabulary knowledge, phonological and morphological awareness measured at age 4 were included as control variables, since the task performance of phonological and morphological awareness showed ceiling effects at age 6. T1-weighted MRI and reading fluency data from the subsample of 79 children were obtained at age 14. This study was approved by an Institutional Review Board of the State Key Laboratory of Cognitive Neuroscience and Learning at Beijing Normal University. Written informed consent was obtained from parents/guardians of the participants for both behavioral tests and MRI session.

### Behavioral Measures

#### Non-verbal IQ

Children were administered sets A and B of the Raven’s Standard Progressive Matrices (Raven et al., 1996) at age 4. They were asked to select one best choice from six options that best fit into the missing part of the original figure. There were 24 trials in total, and one point was awarded for each correct trial.

#### Cross Out

Children were administered this task at ages 6 to 8. The task was from the Woodcock-Johnson Tests of Cognitive Ability (Woodcock and Johnson, 1989). In each trial, there was a row of 20 items. Children had to cross out five of those that were the same as the target item appearing in the beginning of the row. There were 30 trials in total. The stimuli adopted in this task are symbols that are not easily verbally codable (e.g., a square with a horizontal line in it). Children were given 3 min and asked to complete the task as quickly as possible. One point was allocated to each correctly answered trial. We calculated the summed raw (rather than normed) score in 3 min.

#### Visual Matching

This task was from the Woodcock-Johnson Tests of Cognitive Ability (Woodcock and Johnson, 1989). Children were tested from ages 6 to 8. In this task, there were five numbers in each trial arranged in a row. Among these five items, two were identical. Children were asked to circle these two numbers in each trial. They were given 3 min and were required to complete as many trials as possible. There were 60 trials in this task. The stimuli were arranged in an order of increasing difficulty from single-digit numbers to three-digit numbers. Each correctly answered trial was given one point and we used the total number of correct answers (raw, rather than normed) as the score of the task.

#### Phonological Awareness

Children’s phonological awareness was tested using a task of syllable deletion (e.g., Pan et al., 2017) at age 4. In this task, a phrase with two or three syllables was presented to the children orally in a trial. Children were asked to delete one specific syllable from the phrase (e.g., deleting /huo3/ meaning fire from /huo3 che1 zhan4/ meaning train station would be /che1 zhan4/ meaning station). There were 18 items. One point was given for each correct answer.

#### Morphological Awareness

The morphological construction task (Pan et al., 2016) was used to indicate morphological awareness. It was administered at age 4. In this task, children were asked to create new words by combining morphemes they had learned. For example, a flower that is red is called a red flower; thus, a flower that is blue should be called a blue flower. There were 15 trials, and one point was given for each correctly answered trial.

#### Vocabulary Knowledge

Children’s oral vocabulary knowledge was tested at age 4 using a vocabulary definitions task (Song et al., 2015) which was adapted from the vocabulary subset of the Stanford-Binet Intelligence Scale (Thorndike et al., 1986) for Mandarin-speaking children. Children were required to explain the words orally presented to them by a trained experimenter and their responses were rated. For instance, the experimenter asked “What is a kitchen?” The answer of “A room for cooking” would be awarded 2 points, whereas 1 point would be allocated to an answer such as “A place at home.” There were 32 words in the test and the maximum score was 64.

#### Character Recognition

This task, administered at age 11, was used to measure children’s reading accuracy (Pan et al., 2017). There were 150 Chinese characters in the task and children were asked to name each individual character. All characters are typically taught in primary school in Mainland China (Shu et al., 2003). The characters were arranged according to their difficulty, from easy to difficult level. The test terminated when the child failed to name 15 characters consecutively. One point was given to each correctly named character. This task was not timed.

#### Reading Fluency

This task was administered at age 14 to the MRI subsample to measure silent reading fluency (Pan et al., 2017). The teens were asked to silently read sentences and judge whether each sentence was correct (e.g., The sun rises from the west) by marking “✓” or “×” at the end of each sentence. There were in total 100 sentences with increasing length, and the teens were given 3 min to read and rate as many as possible. The total number of characters for all items that were correctly judged was used as a score of silent reading fluency.

### Data Analyses

#### Analyses of Behavioral Measures

Latent growth modeling was applied using Mplus version 7.11 (Muthén and Muthén, 2012) to estimate the initial status (intercepts) and growth rates (slopes) of children’s performance in the Cross Out and Visual Matching tasks. The weights of intercepts were fixed at 1 for all three time-points, and the coefficients of slope were assigned to 0, 1, and 2 at the first, second, and third assessments separately. Maximum likelihood estimation was used to deal with missing values. Multiple regression analysis was then adopted to test the contributions of the intercepts and slopes of Cross Out and Visual Matching to character reading at age 11 in the whole sample, with age, sex, non-verbal IQ, phonological awareness, morphological awareness and oral vocabulary knowledge as control variables using the R (version 3.6.2 64 bit; Ihaka and Gentleman, 1996). Given that only 79 out of the 293 children in our longitudinal sample participated in the MRI session, we also replicated all regression analyses in this subgroup.

#### MRI Data Acquisition

High-resolution T1-weighted images were collected for each child at age 14 on a 3-Tesla MAGNETOM Trio Tim scanner (Siemens AG, Germany) at Beijing Normal University with a 12-channel head coil using the following parameters: 144 sagittal slices; repetition time, 2530 ms; echo time, 3.39 ms; inversion time, 1100 ms; flip angle, 7 degrees; field of view, 256 × 192; voxel size, 1.3 × 1.0 × 1.3 mm^3^. Prior to the formal experiment, children lay supine in a mock scanner to acclimate to the testing environment and noise. During scanning, children were provided with padding to hold their head in place to reduce head motion.

#### MRI Data Preprocessing

FreeSurfer version 5.3.0^1^ was used to extract cortical matrices from the T1-weighted images. First, raw T1-weighted images were visually inspected by experts who were blinded to the information of the participants. Two children were excluded due to head motion artifacts. Then, an automatic pipeline consisting of motion correction, removal of non-brain tissue, intensity normalization, automated Talairach transformation, volumetric structure segmentation, cortical surface reconstruction and parcellation was performed (Dale et al., 1999; Fischl et al., 1999). FreeSurfer reconstructed the cortical surface that was represented by a mesh of triangles (vertices) for each subject. Cortical thickness and cortical surface area were calculated at each vertex. Cortical thickness was defined as the distance between the white and pial surfaces. Cortical surface area was defined as the average area of all the triangles that meet at a given vertex on the white matter surface. The resulting images were resampled to a standard brain, and surface maps were smoothed with a full-width half-maximum of 10 mm.

#### MRI Data Analyses

General linear modeling was used to estimate the relationship between vertex-wise cortical morphometric measures (cortical thickness and surface area) with the developmental parameters of Cross Out, Visual Matching, and character recognition. Age and sex were included as nuisance covariates. The resulting statistical maps were corrected for multiple comparison errors with Monte Carlo simulations implemented in FreeSurfer (10,000 iterations; uncorrected *p*-vertex < 0.01, corrected *p*-cluster < 0.05; Hagler et al., 2006). Significant clusters were reported in Talairach coordinates.

Conjunction analysis was carried out to identify shared neurobiological substrates of character recognition at age 11 and developmental measures of visual skills. To be specific, we created a conjunction map by combining the brain maps corrected for multiple comparison errors in the whole brain analysis. Overlapping areas were defined as the region of interest (ROI) for the subsequent analysis. Because we used cognitive measures collected earlier than MRI data in the whole-brain analysis, here we further tested whether the brain matrices in the overlapping areas were also associated with the concurrent reading abilities at age 14. Considering ceiling effect of the character recognition task in middle school students and the goal of reading acquisition is comprehension, we employed reading fluency task as our literacy measure of interest at age 14. This measure captures word reading accuracy and fluency, as well as comprehension, which is a more comprehensive measure of reading ability at a later stage. Since word reading acts as a subcomponent of reading fluency that also encompasses speed of processing and appropriate expression skills (Klauda and Guthrie, 2008), neural substrates associated with wording reading skill should also correlate with reading fluency performance. Thus, the brain matrices of the overlapping areas in the conjunction analysis were extracted and correlated with the reading fluency measure at age 14.

## Results

Table 1 shows the means, standard deviations, and correlations of all behavioral variables in the entire sample. We then tested the contribution of intercepts and slopes of Visual Matching and Cross Out to reading ability.

**TABLE 1:** Descriptive statistics and correlations among behavioral measures.

**Measures**	***N***	***Mean***	***SD***	***Range***	**1**	**2**	**3**	**4**	**5**	**6**	**7**	**8**	**9**	**10**	**11**	**12**	**13**
1. N-IQ	293	10.42	2.52	5–21	–												
2. CA	293	53.53	3.64	46.36–62.86	0.38***	–											
3. CO-6	292	10.53	3.06	1–22	0.28*	0.31**	–										
4. CO-7	291	15.14	2.85	7–24	0.38***	0.23*	0.34**	–									
5. CO-8	293	17.97	3.47	9–29	0.44***	0.33**	0.41***	0.69***	–								
6. VM-6	293	21.96	5.59	0–41	0.32**	0.33**	0.52***	0.52***	0.50***	–							
7. VM-7	293	29.67	5.22	13–46	0.24*	0.18	0.21	0.63***	0.60***	0.60***	–						
8. VM-8	293	35.18	5.66	17–51	0.30**	0.20	0.24*	0.59***	0.69***	0.53***	0.75***	–					
9. PA-4	293	8.40	4.89	0–15	0.31**	0.19	0.29*	0.19	0.38***	0.33**	0.22	0.36**	–				
10. MA-4	293	10.23	3.25	0–15	0.34**	0.18	0.33**	0.33**	0.43***	0.48***	0.41***	0.41***	0.67***	–			
11. VK-4	293	6.23	3.72	0–20	0.27*	0.21	0.11	0.31**	0.37**	0.23*	0.12	0.14	0.36**	0.42***	–		
12. CR-11	291	123.36	12.30	68–144	0.12	0.11	0.13	0.36**	0.37***	0.44***	0.45***	0.47***	0.43***	0.44***	0.29*	–	
13. RF-14	77	479.42	132.83	114.67–984	0.10	0	0.15	0.43***	0.42***	0.31**	0.41***	0.45***	0.30**	0.34**	0.41***	0.52***	–

N-IQ, Non-verbal IQ; CA, chronological age (in month); CO, Cross Out; VM, Visual Matching; PA, phonological awareness; MA, morphological awareness; VK, vocabulary knowledge; CR, character recognition; RF, reading fluency. The number appended to the measures indicate the age of the test performed. *p < 0.05; **p < 0.01; ***p < 0.001.

### Contributions of Visual Matching and Cross Out to Reading

Table 2 shows that, after controlling for effects of age, sex, non-verbal IQ, phonological awareness, morphological awareness and oral vocabulary knowledge, only the Visual Matching slope significantly predicted character recognition at age 11, suggesting that children with a faster growth rate in the Visual Matching task had better reading performance as compared to those with a slower growth rate. We further replicated the entire analysis in the subsample of the 79 children who participated in the MRI session and observed the same pattern of significance (Supplementary Table S1), suggesting that the subsample results were representative of that in the whole sample. We performed the *post hoc* power analysis for all linear regression models with the WebPower package in R (Zhang and Yuan, 2018) and resultant *post hoc* powers were larger than 0.80 (Cohen, 1988).

**TABLE 2:** Multiple regression analysis for the entire sample.

	**CR-11**		
**Predictors**	**β**	***t*_280_**	***p***
Gender	0.14	1.46	0.145
N-IQ	−0.10	–1.80	0.074
CA	−0.03	–0.57	0.572
PA-4	0.21	3.43	0.001
MA-4	0.19	2.99	0.003
VK-4	0.10	1.84	0.066
CO-INT	0.11	1.40	0.163
CO-SLP	−0.03	–0.44	0.657
VM-INT	0	0.07	0.943
VM-SLP	0.30	3.99	<0.001

*CR, character recognition; CA, chronological age; PA, phonological awareness; MA, morphological awareness; VK, vocabulary knowledge; CO, Cross Out; VM, Visual Matching; INT, intercept; SLP, slope; The number appended to the measures indicate the age of the test.*

### MRI-Related Findings

**FIGURE 1:**
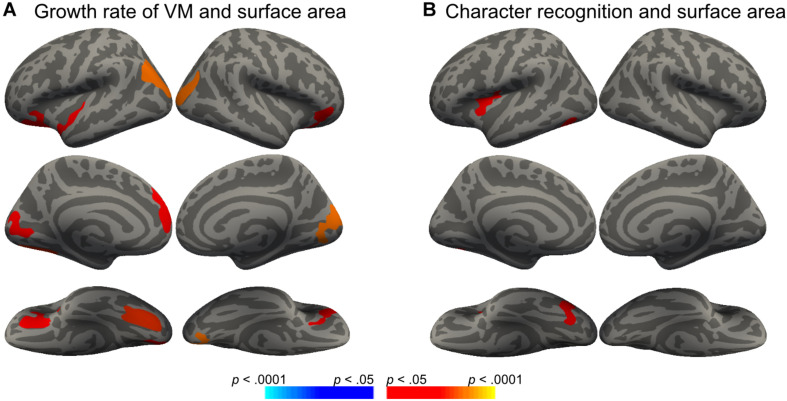
Significant clusters in the vertex-wise whole-brain analyses (Monte Carlo procedure for correction of multiple comparisons, 10000 iterations, vertex-*p* < 0.01, cluster-*p* < 0.05; red = positive correlation, blue = negative correlation). **(A)** Surface areas in eight clusters were positively correlated with the growth rate of Visual Matching (VM-SLP), while age and sex were statistically controlled. **(B)** Surface areas in two clusters were positively correlated with children’s character reading at Grade 5 (CR-11), while age and sex were statistically controlled.

**FIGURE 2:**
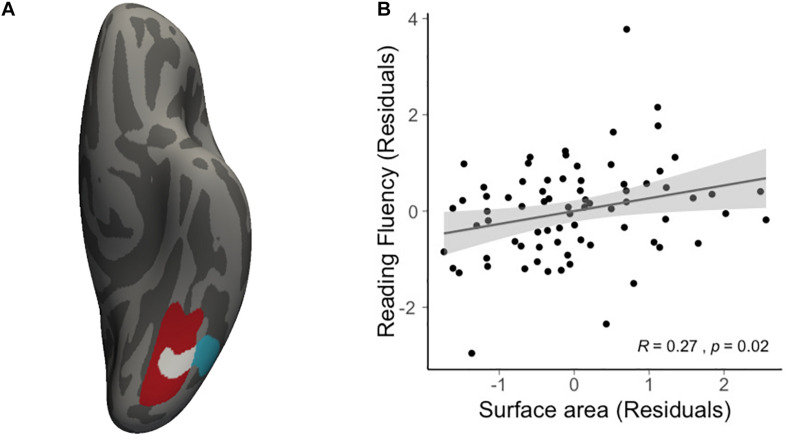
Results of conjunction analysis. **(A)** The spatial overlap (white) of significant clusters obtained from whole brain analyses on the growth rate of VM (red) and character recognition at age 11 (cyan). **(B)** Scatter plot illustrates the positive correlation (*r* = 0.27, *t*_73_ = 2.37, *p* = 0.021) between average cortical surface area of the overlapping region and concurrent reading fluency at age 14, statistically controlling for age, sex and non-verbal IQ.

We then conducted vertex-wise whole-brain regression analyses on cortical thickness and cortical surface area, separately. As predicted, we observed a positive correlation between the Visual Matching slope and the cortical surface area in widespread brain areas including the left fusiform gyrus after the correction for multiple comparisons (Figure 1A and Table 3). Children who developed faster in Visual Matching had a larger cortical surface area in those regions. For character recognition at age 11, two clusters in the left fusiform gyrus and insula (including the pars opercularis) survived under the whole brain multiple comparisons correction (Figure 1B and Table 3). In terms of cortical thickness, no significant clusters were found to be associated with the intercept and slope of Visual Matching, Cross Out, or character recognition at age 11. In the conjunction analysis, spatial overlap of clusters related to the Visual Matching slope and character recognition was identified at the left fusiform gyrus only (Figure 2A; Area: 281 mm^2^; Center of gravity: −34, −70, −14; Talairach coordinates). Moreover, to evaluate the concurrent brain-reading relationship, Pearson’s correlation between cortical surface area of the overlapping area in the left fusiform with concurrent reading fluency was calculated and a significant, positive correlation was observed (Figure 2B, *r* = 0.27, *t*_73_ = 2.37, *p* = 0.021).

**TABLE 3:** Results of a whole brain vertex-wise analysis after correction for multiple comparisons using a cluster-based Monte Carlo procedure (10000 iterations; vertex-*p* < 0.01, cluster-*p* < 0.05) from the subsample of 79 participants.

**Behavior**	**Regions**	**Cluster**		**Talairach Coordinates**		
		**Size**	***p*-value**	***x***	***y***	***z***
***Surface area***						
VM-SLP	LH-inferior parietal	2350.14	<0.001	−40	−76	25
	LH-fusiform	1254.34	<0.001	−29	−74	−5
	LH-superior frontal	1053.00	0.001	−9	52	8
	LH-pericalcarine	996.08	0.002	−9	−75	7
	LH-orbitofrontal	984.81	0.002	−26	16	−20
	LH-superior temporal	578.00	0.044	−42	−2	−19
	RH-inferior parietal	3387.80	<0.001	37	−76	16
	RH-parsorbitalis	733.64	0.013	47	35	−11
CR-11	LH-fusiform	598.40	0.036	−39	−60	−3
	LH-insula	580.62	0.042	−32	6	9
***Cortical thickness:*** No significant results						

*VM-SLP, Slope of Visual Matching; CR-11, character recognition at age 11; LH, left hemisphere; RH, right hemisphere. The unit of cluster size is mm^2^.*

## Discussion

In the present study, we first investigated the independent contributions of two tasks, Cross Out and Visual Matching, to reading performance. Cross Out is a task that involves speeded visual processing and Visual Matching is a task that involves speeded visual processing and implicit phonological processing. Moreover, we further examined the shared cortical morphometry, supporting both the growth of Visual Matching and reading. Results showed that the growth rate of Visual Matching uniquely predicted reading ability. Importantly, an overlapping region in the left fusiform gyrus was related to both the growth rate of Visual Matching and reading.

### Role of Visual Matching

Learning to read involves mapping print to sound. As children start formal education, this mapping becomes increasingly important. The Chinese writing system is well-known for its opaque print-to-sound mappings. Children acquire a large number of such arbitrary mappings between characters and their pronunciations when they enter primary school. Thus, the automaticity of activating phonological representations of the characters is important in learning to read. Previous studies have shown that dyslexic readers are particularly characterized as having deficits involving alphanumeric stimuli rather than symbols that are not easily verbally codable (Ziegler et al., 2010; Pan et al., 2013; Bakos et al., 2017). As for typically developing children, Pan et al. (2020) found that Visual Matching at age 6 predicted reading accuracy at age 7 while Cross Out did not. Our current findings that Visual Matching but not Cross Out predicted reading performance are in agreement with previous findings, suggesting that the ability to map visual information to phonological codes is important in learning to read universally (Ziegler and Goswami, 2005; Rueckl et al., 2015).

While the contrast of Visual Matching and Cross Out underscores the importance of print-to-sound mappings, it should be noted that only the growth rate but not the initial status of Visual Matching predicted reading. The Visual Matching task was administered at ages 6, 7 and 8. The first measurement at age 6 occurred just before children entered primary school, while children were mostly in first and second grades when they were tested at ages 7 and 8. Studies using event-related potential (ERP) have demonstrated that the N1 (or N170) component is larger for words than for symbols when children start learning to read (Maurer et al., 2005). Our findings are in line with these ERP studies, suggesting that the development of efficient, even automatic linking of visual codes to phonological codes is important in the early stage of formal education. This development forms the foundation for subsequent reading development.

### Role of the Left Fusiform Gyrus

The neuroimaging results further confirmed our behavioral findings, that is, left fusiform appears to support both development of print-to-sound mapping skill and character reading. We found that surface area, but not cortical thickness in the left fusiform gyrus, was associated with the growth rate of Visual Matching and subsequent character reading. Moreover, the growth rate of Visual Matching and word reading ability were both linked to the left fusiform gyrus, which was further correlated with more advanced reading abilities. Previous studies have shown that the left fusiform gyrus is closely associated with recognition of word-like stimuli (Dehaene et al., 2010) and it also incorporates both orthographic and phonological information of Chinese characters (Zhao et al., 2016). In particular, increasing activation in the left fusiform gyrus appears to occur in parallel with the acquisition of a correspondence between orthography and phonology in both preschool children (Brem et al., 2010) and adults (Hashimoto and Sakai, 2004). Previous studies with atypically developing children have indicated that both resting-state and task-driven functional connectivity between the left fusiform gyrus and the parietal cortex were decreased in dyslexic readers (Vogel et al., 2014). The current study extended these findings to typically developing children, suggesting that the left fusiform gyrus provides a neural gateway by which to integrate print and sound for typically developing children as well.

It is worth mentioning that, the neuroimaging data in the present study were collected together with the fluency measure at age 14, later than the Visual Matching task and the reading accuracy task which was collected at age ages 6 to 8 and 11, respectively. This allowed us to investigate the long-lasting association between Visual Matching development, reading, and neuroanatomical features from a developmental perspective. Although relevant longitudinal research is scarce, associations between early cognition and brain features measured later have been examined recently (Kremen et al., 2019). Walhovd et al. (2016) reported a high stability in the relationship between the cortical surface area and general cognitive ability throughout life, suggesting that this association might be genetically determined, at least in part. Here, we found that both print-to-sound mapping ability and reading ability were associated with cortical morphometry measured at a later time, suggesting a stable brain-reading correlation in specific brain areas, including the left fusiform gyrus.

### Limitations

There are mainly two limitations in the present study. First, since the neuroimaging data were collected at only one time-point, we were unable to examine the developmental dynamics of cortical morphometry and its relationship with reading acquisition. Second, while multiple cognitive and neural characteristics have been found to be impaired in dyslexia, in support of the multiple deficits model of atypical reading development (Pennington, 2006), we mainly examined visual and phonological processes in the present study. To gain a broader understanding of the relationship between cognitive skills and literacy development, as well as to investigate the underlying mechanisms, future studies should use more neurocognitive measures, multimodal neuroimaging methods, and longitudinal MRI assessments.

## Conclusion

To conclude, the present study highlighted the importance of the visual-verbal conversion components of the Visual Matching task in predicting reading ability. It also highlighted the role of the left fusiform gyrus in the development of such this coding process among typical developing children. Given the differences between Chinese and alphabetic scripts, further studies are needed to further investigate whether the current findings can be generalized to other writing systems including alphabetic scripts.

## Data Availability Statement

The raw data supporting the conclusions of this article will be made available by the authors, without undue reservation.

## Ethics Statement

The studies involving human participants were reviewed and approved by Institutional Review Board of the State Key Laboratory of Cognitive Neuroscience and Learning, Beijing Normal University. Written informed consent to participate in this study was provided by the participants’ legal guardian/next of kin.

## Author Contributions

XC and ZX conceived of the original idea and carried out the data analysis. XC, ZX, and JP developed the theory and wrote the manuscript with the help of CM, PL, and HS. JP and HS supervised the project. All authors provided critical feedback and helped shape the research, analysis, and manuscript.

## Conflict of Interest

The authors declare that the research was conducted in the absence of any commercial or financial relationships that could be construed as a potential conflict of interest.

## Footnotes

1 http://surfer.nmr.mgh.harvard.edu
