# Supplementary material for: Shared Neural Substrates Underlying Reading and Visual Matching: A Longitudinal Investigation
Source: Front Hum Neurosci. 2020 Oct 22;14:567541. doi: 10.3389/fnhum.2020.567541 (PMC7642616; doi:10.3389/fnhum.2020.567541)
Supplement: Supplementary file 1 [file Data_Sheet_1.docx]

Supplementary Material

# Supplementary Figures and Tables

Table S1 shows the results of a regression analysis of the subsample, replicating the findings of the whole sample. This analysis shows that growth rate of Visual Matching predicted character recognition at age 11 with gender, age, nonverbal IQ, phonological awareness, morphological awareness and vocabulary knowledge statistically controlled.

**Table S1** Multiple regression analysis predicting character recognition at age 11 from initial status and growth rate of Visual Matching and Cross Out in the MRI subsample (N=79).

|  | CR-11 | | | |
| --- | --- | --- | --- | --- |
| Predictors | *β* | *t*_68_ | *p* |  |
| gender | 0.19 | 0.97 | 0.334 |  |
| N-IQ | -0.15 | -1.33 | 0.188 |  |
| CA | 0 | 0.02 | 0.981 |  |
| PA-4 | 0.28 | 2.09 | 0.040 |  |
| MA-4 | 0.08 | 0.54 | 0.593 |  |
| VK-4 | 0.12 | 1.02 | 0.310 |  |
| CO-INT | 0.04 | 0.25 | 0.805 |  |
| CO-SLP | -0.07 | -0.57 | 0.570 |  |
| VM-INT | 0.12 | 1.03 | 0.305 |  |
| VM-SLP | 0.34 | 2.28 | 0.026 |  |

*Note*: CR = character recognition; N-IQ = Nonverbal IQ; CA = chronological age; PA = phonological awareness; MA = morphological awareness; VK = vocabulary knowledge; CO = Cross Out; VM = Visual Matching; INT = intercept; SLP = slope; The number appended to the each measure indicates the age at which the test was administered.

As requested by reviewers, we also conducted the same regression analysis for the reading fluency measure. Table S2 shows that the growth rate of Visual Matching predicted reading fluency at age 11 in the entire sample and at age 14 in the MRI subsample, respectively.

**Table S2** Multiple regression analysis predicting reading fluency from the initial status and growth rate of Visual Matching and Cross Out in the entire sample (N=293) and in the MRI sample (N = 79), respectively.

|  | RF-11 | | |  | RF-14 | | |
| --- | --- | --- | --- | --- | --- | --- | --- |
| Predictors | *β* | *t*_280_ | *p* |  | *β* | *t*_66_ | *p* |
| gender | 0.29 | 3.1 | 0.002 |  | 0.26 | 1.33 | 0.188 |
| N-IQ | -0.02 | -0.42 | 0.678 |  | -0.12 | -1.09 | 0.281 |
| CA | -0.16 | -3.21 | 0.001 |  | -0.17 | -1.62 | 0.111 |
| PA-4 | 0.1 | 1.75 | 0.082 |  | 0.1 | 0.73 | 0.469 |
| MA-4 | 0.04 | 0.68 | 0.5 |  | 0 | -0.01 | 0.99 |
| VK-4 | 0.26 | 4.92 | <0.001 |  | 0.34 | 2.91 | 0.005 |
| CO-INT | 0.07 | 0.91 | 0.363 |  | 0.11 | 0.67 | 0.504 |
| CO_SLP | 0 | 0.07 | 0.946 |  | 0.05 | 0.43 | 0.665 |
| VM-INT | -0.03 | -0.59 | 0.553 |  | -0.04 | -0.31 | 0.756 |
| VM-SLP | 0.42 | 5.84 | <0.001 |  | 0.36 | 2.42 | 0.018 |

*Note*: RF = reading fluency; N-IQ = Nonverbal IQ; CA = chronological age; PA = phonological awareness; MA = morphological awareness; VK = vocabulary knowledge; CO = Cross Out; VM = Visual Matching; INT = intercept; SLP = slope; The number appended to each measure indicates the age at which the test was administered.
